# Pharmacognosy and Antioxidant Activity of Pruned Leaves from the Unexplored *Olea europaea* L. ‘Lavagnina’ (Liguria, Italy)

**DOI:** 10.3390/molecules30173605

**Published:** 2025-09-03

**Authors:** Federica Betuzzi, Paola Malaspina, Flavio Polito, Giovanni Bottino, Vincenzo De Feo, Laura De Martino, Laura Cornara

**Affiliations:** 1 Department of Earth, Environment and Life Sciences (DISTAV), University of Genova, Corso Europa 26, 16132 Genova, Italy; federica.betuzzi@edu.unige.it; 2 Department of Pharmacy, University of Salerno, Via Giovanni Paolo II 132, 84084 Fisciano, Italy; fpolito@unisa.it (F.P.); defeo@unisa.it (V.D.F.); ldemartino@unisa.it (L.D.M.); 3 Consorzio di Tutela del Basilico Genovese DOP, Salita Santa Caterina 50-52R, 16123 Genova, Italy; gianni.bottino@basilicogenovese.it

**Keywords:** olive by-products, leaf micro-morphology and anatomy, phytochemistry, antioxidant properties

## Abstract

*Olea europaea* L. ‘Lavagnina’ is cultivated in the Eastern Ligurian coast (Italy), and during the pruning process a huge amount of pruning residues is produced. This by-product is generally disposed of by burning, despite still containing bioactive compounds. In particular, olive leaves are indeed rich in secondary metabolites, which can vary both in quality and quantity in relation to the cultivar considered and the area of cultivation. For this reason, we aimed to carry out a pharmacognostic study of the pruned leaves of the unexplored local cultivar ‘Lavagnina’, evaluating the possibility of reusing this by-product for new health applications. The micromorphological characterization was conducted by light and scanning electron microscopy. ‘Lavagnina’ leaf was micromorphologically similar to that of other olive cultivars; however, it differed in terms of midrib structure. Leaf extracts were obtained using solvents of increasing polarity (petroleum ether, chloroform, methanol) and the food-grade solvent, 70% ethanol. A high antioxidant activity was found only for the methanolic (ME) and hydroalcoholic (HAE) extracts, and, therefore, they were then characterized from a phytochemical point of view by LC-ESI-HR-MS. Such analysis allowed the identification of secondary metabolites belonging mainly to secoiridoids, flavonoids, and iridoids. Overall, the HAE had the highest antioxidant activity (17.3 ± 0.6 μg/mL), and it is, therefore, the best candidate for health applications related to a protective effect on a variety of inflammation-related diseases, also considering that inflammation may play a role in cancer progression.

## 1. Introduction

In the Mediterranean basin, the olive tree (*Olea europaea* L. subsp. *europaea*) is one of the most important agricultural crops [1], with Spain, Italy, and Greece as the leading worldwide producers of olive oil and table olives [2].

In Liguria Region (Italy), the olive growing area is small (about 2000 hectares), but the peculiar pedo-climatic characteristics of the environment and the traditional cultivation methods allow for obtaining a typical extra-virgin olive oil (EVO) certified by the Protected Designation of Origin (PDO). This product is highly valued all over the world [3,4], placing Liguria among the Italian regions excelling in the art of oil-making.

The main cultivars of *O. europaea* used to make EVO in Liguria are ‘Taggiasca’ and ‘Lavagnina’, both of which owe their name to two towns, Taggia (Imperia province) and Lavagna (Genoa province), respectively. In particular, the ‘Lavagnina’ olive is widespread in the East of Liguria (Province of Genoa, Gulf of Tigullio), and it is used to produce the PDO oil “Riviera Ligure—Riviera di Levante”. According to the PDO Product Specifications [5], ‘Lavagnina’ olives are used alone or mixed with small quantities of other cultivars, such as ‘Razzola’ and ‘Pignola’.

‘Lavagnina’ was already mentioned by G. Gallesio (1817–1839), an agronomist and botanist from Savona (Liguria), in his *Pomona Italiana*, a treatise on Italian fruit trees [6]. This early documentation highlights the long-standing presence and agricultural relevance of this cultivar in Liguria. Despite this, ‘Lavagnina’ has been little studied. The only reference to *O. europaea* ‘Lavagnina’ appears in an Italian publication edited by Regione Liguria [7], which reports the phytochemical differences among virgin olive oils obtained from various Ligurian cultivars of *O. europaea*. A few other works mentioning ‘Lavagnina’ focus on the molecular identification of cultivars [8,9].

In light of its historical and economic significance, investigating and safeguarding ‘Lavagnina’ is essential to preserve the Ligurian olive-growing heritage and maintain the PDO olive oil production.

Nowadays, the cultivation of ‘Lavagnina’ in Eastern Liguria spreads over a few hectares, but olive farming and oil production generate numerous by-products, such as leaves and pomace. In particular, the maintenance of a medium-sized olive grove requires pruning operations to maintain tree health, resulting in a huge amount of leafy branches per year. These pruning residues are sometimes chipped and spread over the soil to reduce erosion but more often are treated as waste and disposed of by burning. A considerable amount of olive leaves is also discarded in the first phase of the oil-making process, when leaves are separated from olives (about 5 kg of leaves per quintal of olives).

Pomace (“sansa”) and fragments of kernels (“nocciolino”) in Eastern Liguria are already valorised: the first is used to produce a low-value oil, while kernels serve as an energy source for heating. In contrast, although they contain a significantly higher percentage of polyphenols than olives or oil, pruned leaves remain underutilized. These compounds could be extracted and used for applications in the medicinal, nutraceutical, and cosmetic sectors [10,11,12,13]. So, exploiting pruning residues of ‘Lavagnina’ would allow the local small companies to open up to new sources of income while promoting a more sustainable olive oil supply chain.

Olive leaves represent a natural source of bioactive compounds with therapeutic effects on health. Indeed, their extracts have been exploited in traditional medicine for centuries, with the first attested use for the treatment of fever, cough, and urinary problems in ancient Egypt [14,15]. Several scientific works have described the ethnobotanical uses of olive leaf preparations in Italy [16] and references therein, and in other Mediterranean countries [17,18], mainly in the form of decoction, for the treatment of various diseases, such as hypertension, stomach problems, kidney stones, hypercholesterolemia, and diabetes.

Many laboratory tests, both in vitro and in vivo, have demonstrated the beneficial properties of olive leaves, including antihypertensive, anticarcinogenic, hypoglycemic, antimicrobial, and hypocholesterolemic effects [11]. All these positive activities can be at least partly ascribed to the antioxidative power of polyphenols [1], such as: (a) secoiridoids (oleuropein and derivatives), (b) flavonoids (apigenin, quercetin, luteolin, luteolin-7-O-glucoside), and (c) simple phenols (hydroxytyrosol and tyrosol, caffeic and vanillic acids) [19].

The qualitative and quantitative phenolic composition of olive leaves can vary significantly according to both genetic and climatic/geographical factors [20,21], highlighting the importance of studying local cultivars.

Considering this, the overall aim of our work was to investigate and valorise the pruned leaves of the ‘Lavagnina’ cultivar growing in Eastern Liguria, to evaluate the potential for recovering bioactive compounds for healthy applications, from a circular economy perspective.

From a scientific point of view, our goal was to chemically characterize pruned leaf extracts and assess their antioxidant activities. In addition, to complete the pharmacognostic study of ‘Lavagnina’, and as a quality control for future applications of the pruned biomasses, we also carried out a morphological and anatomical characterization of the leaves by Light and Scanning Electron Microscopy to highlight their distinctive features, which can help to ensure the purity and safety of this by-product.

From an economic and regulatory perspective, our in-depth study of this unexplored typical Ligurian cultivar is important to support its peculiar intrinsic characteristics that derive from the set of terroir, genetics, and agricultural practices [22]. The whole of the data collected could represent a solid scientific basis that may contribute to a potential PDO or PGI (Protected Geographical Indication) recognition of ‘Lavagnina’ olive.

## 2. Results

### 2.1. Micro-Morphological and Anatomical Analysis

**Figure 1:**
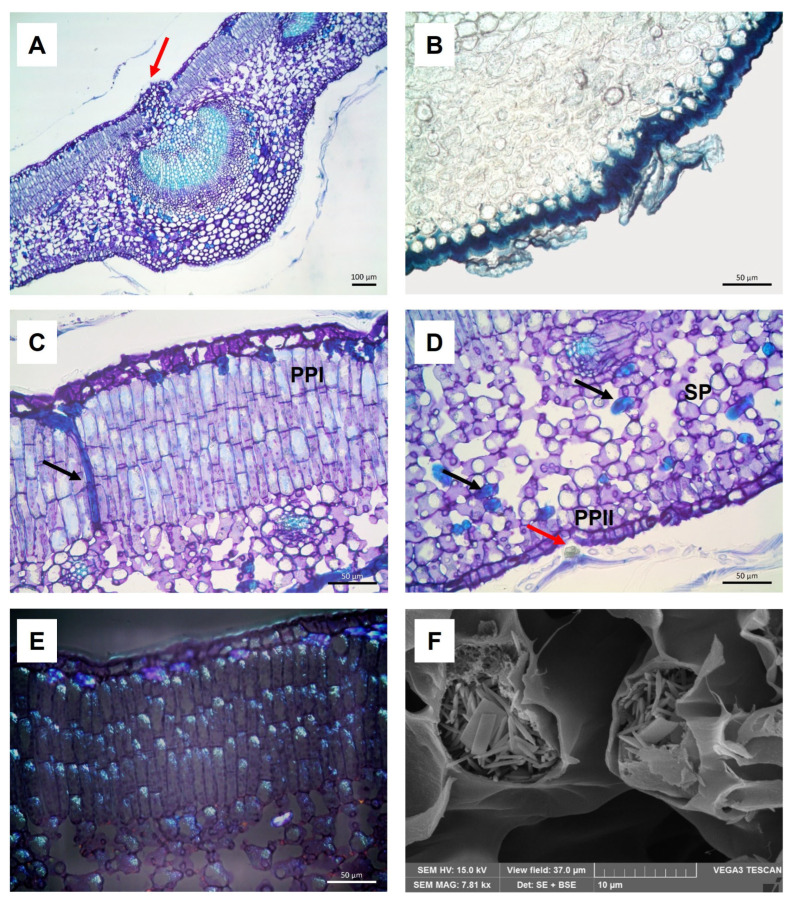
(**A**–**E**) Light Microscopy micrographs of *Olea europaea* L. ‘Lavagnina’ leaf sections embedded in Technovit 7100 and stained with TBO (**A**,**C**,**D**) or Sudan Black (**B**) or observed under polarized light (**E**): (**A**) midrib with a small protrusion made up of mechanical tissues (arrow); (**B**) thick abaxial cuticle and scutiform trichomes; (**C**) longitudinally oriented trichosclereid through the PPI (arrow); (**D**) SP, PPII, tangentially oriented trichosclereids (black arrows) and a scutiform trichome with phenolic content in its stalk cell (red arrow); (**E**) small birefringent calcium oxalate crystals both in PPI and SP. Bar = 100 μm (**A**), Bars = 50 μm (**B**–**E**). (**F**) SEM micrograph of leaf section, showing small calcium oxalate crystals, mainly raphides, in the SP.

The leaf was iso-bilateral and hypostomatic, showing collenchyma layers and scattered sclerenchyma fibers under the epidermis both above and below the midvein (Figure 1A). In particular, in the adaxial surface, these tissues gave rise to a characteristic small protrusion (Figure 1A, arrow). A thick cuticle was present on both surfaces, especially on the abaxial one, as highlighted by the staining with Sudan Black (Figure 1B).

The mesophyll was divided into: (a) three to four layers of palisade parenchyma I (PPI) under the adaxial epidermis, rich in phenolics as indicated by the light blue staining with TBO (Figure 1C); (b) spongy parenchyma (SP) and (c) one layer of palisade parenchyma II (PPII) close to the abaxial epidermis (Figure 1D). The cells of PPII were short, irregularly shaped, and not well distributed compared to the cells of PPI.

Under the adaxial epidermis and among the spongy cells, several filiform sclereids (trichosclereids) were observed, longitudinally oriented in PPI (Figure 1C, arrow) and tangentially oriented in SP (Figure 1D, black arrows).

In both PPI and SP, numerous small calcium oxalate crystals, mainly in the form of raphides, were found. They appeared well visible under polarized light (Figure 1E) and more detailed by Scanning Electron Microscope (SEM) (Figure 1F).

Numerous non-glandular scutiform trichomes were observed on both surfaces (Figure 1B–D), and the histochemical analysis showed in their unicellular stalk cell the presence of phenolic compounds, as highlighted by the green staining with TBO (Figure 1D, red arrow).

**Figure 2:**
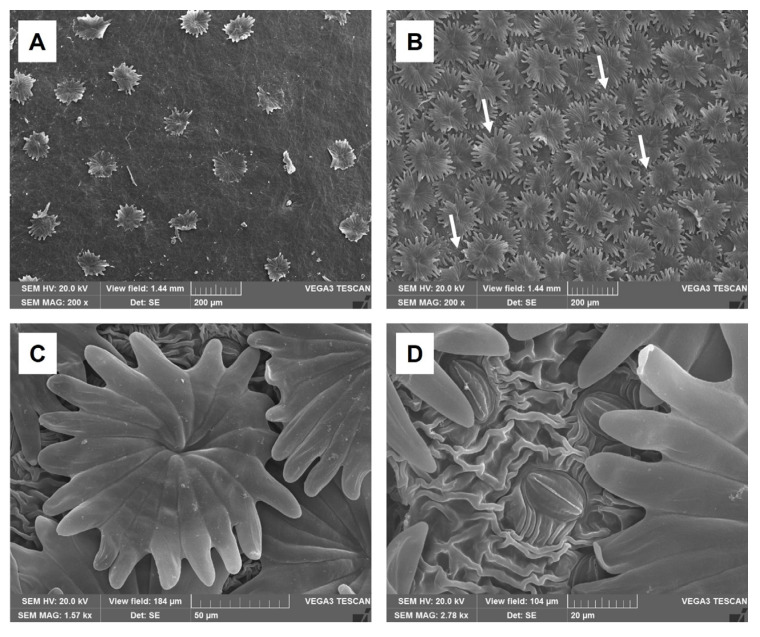
SEM micrographs of *Olea europaea* L. ‘Lavagnina’: (**A**) leaf adaxial surface showing sparse scutiform trichomes; (**B**) leaf abaxial surface with numerous not tightly overlapping trichomes, revealing small areas of the underlying epidermis (arrows); (**C**) magnification of a scutiform trichome on the abaxial surface; (**D**) detail of the striated cuticle and of some stomata on the abaxial surface.

Moreover, SEM investigation revealed that these trichomes were sparse on the adaxial epidermis (Figure 2A), while on the abaxial epidermis they were densely distributed but did not strictly overlap (Figure 2B, arrows). Thus, in some areas, it was possible to observe the underlying striate cuticle and stomata (Figure 2C,D).

All these anatomical traits of olive leaf are conserved among cultivars; however, the structure of the midrib can slightly differ, as shown in Figure S1, where ‘Lavagnina’ (A) is compared to other two cultivars, that are ‘Taggiasca’ (B) and ‘Leccino’ (C).

### 2.2. Extraction

The extraction yield (%) of petroleum ether extract (PEE), chloroformic extract (CHE), methanolic extract (ME) and hydroalcoholic extract (HAE) was 0.5, 3.3, 3.2, and 7.2, respectively.

### 2.3. Antioxidant Activity

The antioxidant activity of the extracts evaluated by DPPH, FRAP, and ABTS assays is presented in Table 1.

The PEE did not exhibit any antioxidant activity. On the contrary, CHE, ME, and HAE had some antioxidant activity in all three tests; nevertheless, it was lower with respect to the standards. The HAE showed a significantly higher activity (*p* < 0.05) than the other extracts only in the DPPH test, with an IC_50_ value of 17.3 ± 0.6 μg/mL. The least active extract was the CHE (IC_50_: 79.4 ± 0.7 μg/mL). Instead, considering the other two tests, all extracts showed an excellent antioxidant activity.

### 2.4. Chemical Analysis

**Figure 3:**
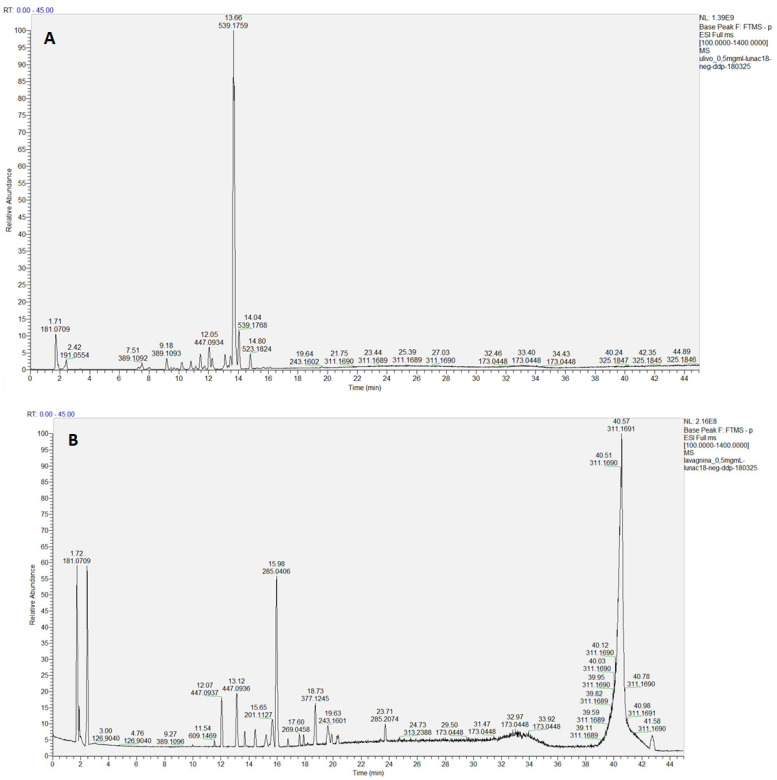
Full scan LC-MS chromatograms (negative ion HRESIMS) of ME (**A**) and HAE (**B**) of *Olea europaea* L. ‘Lavagnina’.

Being the methanolic (ME) and the hydroalcoholic (HAE) extracts the more biologically active, they were analyzed by LC-HRESIMS/MS (Figure 3).

**Figure 4:**
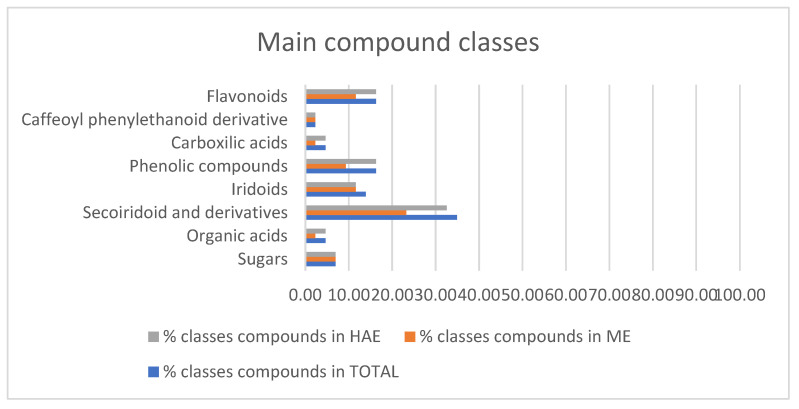
The main compound classes and their distribution (%) between ME and HAE.

Overall, 43 components (Table 2) were identified, belonging to several representative classes of constituents, mainly secoiridoids and their derivatives (15 compounds, peaks 5, 8, 11, 16–18, 20, 24, 26, 28, 31–34, 40) and flavonoids (7 compounds, peaks 27, 29–30, 36–39) (Figure 4). Most metabolites were common to both extracts (e.g., mannitol, quinic acid, hydroxytyrosol and its glycosides, oleoside, oleuropein, rutin, luteolin, quercetin), but some compounds were specific to each extract, e.g., aucubin and ligstroside only in ME, gluconic acid and vanilloside only in HAE (Table 2).

Compound **1** appeared at tR = 1.72 min and yielded a precursor ion [M-H]^−^ at *m*/*z* 181.0709, attributed to sugar mannitol, previously reported by Toumi and coworkers [23] in *O. europaea* roots. MS2 fragmentations revealed characteristic fragments at *m*/*z* 163.0604 and 119.0339, corresponding to the loss of water molecules (-H_2_O), as also reported from the same Authors.

The compound **4** (C_8_H_18_O_8_) was identified in both extracts and could be tentatively annotated as a sugar derivative, as 3-deoxy-D-manno-octulosonate. The major fragment in MS2 spectrum for this compound appeared at *m*/*z* 87.0075, already previously reported in *O. europaea* extract by Serrano-García and coworkers [24].

Another sugar derivative was identified as 1,5-anhydroxylitol, already found in leaves of *O. europaea* [25], which gave a precursor ion [M-H]^−^ at *m*/*z* 259.0825: the identification was confirmed by MS2 analysis, which produced the main fragments at *m*/*z* 217.0701, 145.0494, and 115.039 [25].

The compounds **2** and **3** were organic acid derivatives: the compound **2** gave a [M-H]^−^ ion at *m*/*z* 195.0503, corresponding to the deprotonated molecular form of gluconic acid. The substance, already reported in ethanolic leaf extract of *O. europaea* [26], gave MS2 fragments at *m*/*z* 177.0398, 129.0183, 99.0075 and 75.0075, attributable to an organic acid, as previously reported [27,28]. The compounds **3** was identified as quinic acid, previously reported in *O. europaea* [29]. Also in these case, MS2 fragmentations revealed characteristic fragments at *m*/*z* 173.0445 [M-H_2_O-H]^−^, 127.0388 [M-2H_2_O-CO-H]^−^, 111.0439 [M-2H_2_O-CO_2_-H]^−^, 93.0334 [M-3H2O-CO2-H)^−^, 85.0283 [M-H_2_O-2CO_2_-H]^−^ [29,30].

The peak 5 was attributed to a secoiridoid and identified as 1-b-D-glucopyranosyl acyclodihydroelenolic acid, previously reported in a by-product of olive oil extraction by Rubio-Senent and coworkers [31].

The peaks 8, 11, 16, 17, 18, 20, 24, 25, 26, 28, 31, 32, 33, 34 and 40 were also attributed to the class of secoiridoids and their derivatives. In fact, compound **8** gave a [M-H]^−^ ion at *m*/*z* 389.1092 and was attributed to oleoside. Its MS2 spectra exhibited a fragment ion peak at *m*/*z* 227, related to the loss of a hexose moiety (162 Da). A fragment ion peak at *m*/*z* 183 indicated a subsequent loss of CO_2_. This fragmentation pathway was previously reported in olive leaf extracts by Kabbash et al. [32].

The peak 11 was attributed to loganic acid, that presented deprotonated [M-H]^−^ ion at *m*/*z* 375.1301. The compound generated the ESI-MS2 ions at *m*/*z* 151 [M−Glc−H_2_O–CO_2_-H]^−^ and *m*/*z* 125 [M−Glc−2CO_2_-H]^−^: the same fragmentation pathway was previously reported [33].

The compound **16**, present only in the HAE, was attributed to a derivative of oleoside, oleoside methyl ester, which gave a precursor deprotonated ion [M-H]^−^ at *m*/*z* 421.1724: the MS2 analysis, with the presence of fragments at *m*/*z* 151.075 and 115.0388, was in agreement to Ammar et al. [27].

Compound **17**, only present in the HAE, was recognized as a secoiridoid: it showed a precursor ion [M-H]^−^ at *m*/*z* 257.1032, having a molecular formula C_12_H_18_O_6_. The MS/MS spectrum formed fragment ions at *m*/*z* 213.1130 [M-CO_2_-H]^−^ and 151.0758 [M-C_3_H_6_O_4_-H]^−^, compatible with 3-hydroxymethyl-2,3-dihydro-5-(methoxycarbonyl)-2-methyl-2Hpyran-4-acetic methyl ester, previously reported in olive oil by products [31].

The secoiridoids 18 and 20, identified in both extracts, were, respectively, recognized as elenolic acid dihexoside isomer (that gave a [M-H]^−^ ion at *m*/*z* 565.1779) and acyclodihydroelenolic acid (that gave a [M-H]^−^ ion at *m*/*z* 245.103). Both compounds were previously reported in the literature by Fayek and coworkers [34] in olive leaf metabolome. Acyclodihydroelenolic acid furnished a MS2 spectra with the main fragments at *m*/*z* 183.1020, a characteristic of secoiridoid compounds due to the loss of H_2_O and COOH, as already reported by the same authors.

Compound **24** gave a [M-H]^−^ ion at *m*/*z* 403.1250 and was attributed to oleoside methyl ester. Ventura and coworkers [35] reported the presence of this compound in the extract of olive leaves. The MS2 analysis confirmed the presence of *m*/*z* product ions at 223.0613, 181.0501, 179.0702, and 101.0233, as already reported in the literature [24,35]. Specifically, the *m*/*z* 223.0613 corresponded to dehydrated elenolic acid and it is due to the loss of an hexose from the molecule [28,36].

The peaks 26 and 28 were attributed, respectively, to demethyl oleuropein and to 10-hydroxyoleuropein, that presented deprotonated [M-H]^−^ ions at *m*/*z* 525.1621 and 555.1727: the major fragments in MS/MS spectrum of demethyl oleuropein appeared at *m*/*z* 389.1088, 209.0452, 195.0656, 165.0550, and 121.0649. The first major fragment was due to the loss of dehydrated 3-hydroxy-tyrosol; the other fragments were due to the loss of a dehydrated glucose moiety and to multiple fragmentations which caused the break of aglycone molecule [15]. The same MS2 analysis was previously reported by Tarchi and coworkers [37]. The MS2 analysis of 10-hydroxyoleuropein revealed major fragments at *m*/*z* 223.0609, corresponding to sequential losses of H_2_O, dihydroxystyrene and glucose [38]. The substance was previously found in olive tree leaves [39].

The peaks 31–34 were, respectively, identified as 2′′-methoxyoleuropein (at *m*/*z* 569.1882), oleuropein (at *m*/*z* 539.1759), lucidumoside C (at *m*/*z* 583.2039), and ligstroside (deoxy oleuropein—at *m*/*z* 523.1826). The latest identified secoiridoid was oleuropein aglycone, that gave deprotonated ion [M-H]^−^ at *m*/*z* 377.1245, with a retention time of 18.73 min. The MS2 analysis of the above compounds was in agreement with literature. So, the MS2 spectra of the compound 2′′-methoxyoleuropein revealed the fragment at *m*/*z* 403,1241, which is due to the cleavage of the phenolic moiety (166 amu), and it could undergo an elimination of the glucose moiety (fragment at *m*/*z* 223.0610), as already reported in the literature [40,41]. The MS2 analysis of oleuropein, having a molecular formula C_25_H_32_O_13_, produced the fragment at *m*/*z* 377.1253 by the loss of a glucosyl moiety: the fragment ion at *m*/*z* 307.0826 was explained by the loss of a C_4_H_6_O from the fragment 377.1253, while the fragment at *m*/*z* 275.0561 derived from the loss of CH_3_OH from the fragment at *m*/*z* 307.0826. The same fragmentation was previously reported [40,41]. In the MS/MS spectrum of lucidumoside C, found in both extracts, the characteristic fragment ions of [M-C_10_H_12_O_3_-H]^−^ at *m*/*z* 403.1261 and of [M-M-C_10_H_12_O_3_-Glu-H]^−^ at *m*/*z* 223.0610, were observed in agreement with Serrano-García and coworkers [24]. As also reported by Quirantes-Piné and coworkers [28], the first fragment was due to cleavage of the phenolic moiety, after which it could undergo elimination of the glucose moiety (fragment 223.0610). Ligstroside, present only in the ME, exhibited a characteristic MS/MS spectrum already reported in the literature: the fragment ions at *m*/*z* 361.1298, and 291.0877 could be attributed to the loss of glycosil moiety (162 uma), and of a C_4_H_6_O group. The reported fragmentation pathway was in agreement with literature [42].

Oleuropein aglycone, found only in the HAE, presented the molecular formula C_19_H_22_O_8_: the product ion scan of the deprotonated molecule formed characteristic fragment ions at *m*/*z* 307.0825 and 275.0571, already reported in the literature [27,39].

The compound **6**, identified as a hydroxylated product of the dialdehydic form of decarboxymethyl-elenolic acid, was the first eluted compound belonging to the class of iridoids: it appeared at tR =7.38 min and yielded a precursor ion [M-H]^−^ at *m*/*z* 199.0608. The compound was only identified in the HAE and produced a MS/MS spectrum with fragment ions at 155.0705, and 111.0804, due to the loss of one and two CO_2_ molecules, respectively. Recently, López-Salas and co-workers [43] reported the same compound in olive leaf; the same MS2 spectra were found in the literature [44,45].

The peaks 12, 13, 15, 21 and 23 were also attributed to iridoids. Compound **12** gave a [M-H]^−^ ion at *m*/*z* 345.1195 and was attributed to aucubin. Do and coworkers [46] reported the presence of this compound in various plants, like plantago, vervein, valerian, gentian, and also olive tree. The MS/MS analysis revealed the main fragments at 183.0656 and 165.0552, due to the loss of a glycosil moiety (162 uma) and of H_2_O, respectively; this fragmentation pathway agrees with the literature [47]. The peaks 13, 15, 21 and 23 were attributed, respectively, to the aldehydic form of decarboxymethyl elenolic acid, 7-deoxyloganic acid, loganin, and lamiol, and presented deprotonated [M-H]^−^ ions at *m*/*z* 215.0922, 359.1353, 389.1459, and 377.1459, respectively. The major fragments in MS/MS spectrum of aldehydic form of decarboxymethyl elenolic acid appeared at *m*/*z* 171.1020, and 153.0912, due to the loss of CO_2_ and H_2_O, respectively. Previously, Lozano-Sanchez et al. [48] and Ribeiro et al. [49] reported the same compound in olive by-products. As reported by Ammar and coworkers [27], the fragmentation pathway of 7-deoxyloganic acid with the characteristic ion at *m*/*z* 197.0814, due to the loss of a glycosyl moiety, confirmed the identification. The MS2 analysis of loganin, an iridoid with molecular formula of C_17_H_26_O_10_, with the presence of fragments at *m*/*z* 345.1531, 119,0343, and 101, 0233 was in agreement with literature [27,50]. Finally, lamiol produced a MS/MS spectrum with main fragments at *m*/*z* 197.0815, 153.0913, due to the consequential losses of glucose moiety (180 uma), and of a CO_2_ molecule, respectively [51].

The peak 7, with a precursor deprotonated ion [M-H]^−^ at *m*/*z* 315.1091, was attributed to hydroxytyrosol glucoside, already found in olive leaf extracts [35,52]: the corresponding MS/MS spectrum was dominated by the peak of deprotonated hydroxytyrosol (*m*/*z* 153.0549), due to the cleavage of the glycosyl moiety and by the peak at 123.0441, resulting from the loss of the CH_2_OH and CHO groups from the hydroxytyrosol original structure. These two fragments are characteristic for the identification of any conjugated hydroxytyrosol present in olive tree organs. The same fragmentation has been previously reported [53].

The peaks 9–10 were attributed to phenols and were found in both extracts: the compound **9** was identified as hydroxytyrosol-diglucoside, which gave a precursor deprotonated ion [M-H]^−^ at *m*/*z* 477.1620. In addition, this conjugated hydroxytyrosol gave a MS/MS spectrum dominated by the fragments 153.0549 and 123.0440, as previously reported [53].

Compound **35**, recognized as sebacic acid, with molecular formula C_10_H_18_O_4_, is a simple phenol, previously found in olive extract [54]: the MS/MS analysis was dominated by the fragments 183.1020 and 157.1226, resulting from the loss of H_2_O and CO_2_ molecules, respectively.

The phenolic shikimic acid (compound **41**) was identified at Rt 22.34 min, with a precursor deprotonated ion [M-H]^−^ at *m*/*z* 173.0448: the MS/MS spectrum revealed the presence of main fragments due to the loss of H_2_O (*m*/*z* = 155.0342), CO (*m*/*z* = 145.0496), CO_2_ (*m*/*z* = 129.0547). This compound was previously reported in an extract from olive pomace [53].

Compound **14** and compound **22** are two phenolic aldehydic substances, also linked to the phenolic group: compound **14**, with a precursor deprotonated ion [M-H]^−^ at *m*/*z* 313.0933 was recognized as vanilloside, known also as glucovanillin. The compound, that is the glucoside of vanillin, gave a MS2 spectrum characterized by the fragment ions at 151.0393, and 123.0441, consistent with the leak of a glucose moiety and to the subsequent loss of CH_2_O group, respectively [55,56]. Compound **22**, correlated to the previous compound, was identified as vanillin, a phenolic aldehyde, with the molecular formula C_8_H_8_O_3_: it gave a precursor ion [M-H]^−^ at *m*/*z* 151.0392. The compound was previously reported in olive leaves [27,28,34]. This assignment was supported by the fragment ions produced in MS/MS spectra: the ion, at *m*/*z* 123.0449, was yielded from the loss of the carbonyl group [28].

The compounds **19** and **42** were attributed to two carboxylic acids: specifically, the peak 19, with a precursor deprotonated ion [M-H]^−^ at *m*/*z* 345.1558, was attributed to 1,1,12,12-dodecanetetracarboxylic acid, acompound that was previously reported in olive leaf extract [54]. The peak 42 was attributed to hexadecanedioic acid, a dicarboxylic acid which gave a deprotonated molecular ion at *m*/*z* 285.2074, previously reported in oil mill waste by Marra and coworkers [57]. The MS2 analysis of the compound yielded a main fragment at *m*/*z* 267.1969, previously reported [58].

Compound **25** was attributed to decaffeoylverbascoside, which gave a deprotonated molecular ion at *m*/*z* 461.1672: the MS2 analysis revealed the presence of the main fragment at 113.0233, previously reported in the literature [27,37].

The peaks 27, 29, 30, 36–39, were attributed to compounds belonging to the class of flavonoids. Compounds **27**, **29**, and **37** gave [M-H]^-^ ions at *m*/*z* 609.1468, *m*/*z* 593.1520 and, *m*/*z* 301.0358, which were attributed to rutin (quercetin 3-O-rutinoside), luteolin-7-O-rutinoside, and quercetin, respectively. The analysis of the MS2 spectra showed a fragment at *m*/*z* 301.0351 for 27, and a fragment at *m*/*z* 285.0407 for 29, both consistent with the lack of a rutinoside moiety (308 uma). MS2 fragmentations of the two isomers, in agreement with literature [37] revealed other characteristic fragments at *m*/*z* 300.0279, 178.9981, 151.0028 for rutin, and 447.0978, for luteolin-7-O- rutinoside, respectively. Quercetin showed a MS/MS spectrum with main fragments at 273.0406, 178.9980, and 151.0029 [59]: the latest peak was generated after a Retro-Diels-Alder (RDA) cleavage of the A ring [60].

The peaks 30 and 36 were attributed to luteolin-O-hexoside isomer and luteolin, with a precursor deprotonated ion [M-H]^−^ at *m*/*z* 447.0936 and 285.2074, respectively. This assignment was supported by the fragment ions produced in MS/MS spectra: for luteolin-O-hexoside isomer, the ion at *m*/*z* 285.0407 revealed a cleavage of the sugar (−162 *m*/*z*) releasing the aglycone form [24]. The MS2 fragmentation of luteolin, with the main fragments at 199.0395, 151.0029, 133.0283, was in agreement with literature [24]: the [M-H]^-^ ion can undergo a RDA fragmentation through the heterocyclic ring, resulting in the formation of product ions with *m*/*z* 151.0029 or 133.0283, the latter being the base peak, corresponding to ^1,3^A and ^1,3^B, respectively [61]. Moreover, the ion with *m*/*z* 175.0392 could correspond to [M-C_3_O_2_-C_2_H_2_O-H]^−^, as already reported [62].

The compounds **38** and **39** were identified as apigenin and diosmetin, only present in the HAE, with the molecular formula C_15_H_10_O_5_ and C_16_H_12_O_6,_ respectively. Both compounds were previously reported by Fayek and coworkers [34]. The MS2 analysis revealed the presence of the main fragments at 151.0027 and 117.0332, generated after RDA fragmentation of the [M-H]^−^, which can be represented as ^1,3^A and ^1,3^B, respectively. The fragment at 107.0127 was due to the loss of CO_2_ from the ^1,3^A ring. On the other hand, diosmetin had a different fragmentation pathway. The most abundant product ion was at *m*/*z* 284.0329 corresponding to the loss of a methyl group. Subsequent loss of CO involving the formation of a five-membered ring led to the ion with *m*/*z* 256.0369 [61].

## 3. Discussion

In Liguria, due to the sloping nature of the land, olive trees are placed on terraces supported by dry stone walls, reducing the risk of landslides and erosion and, at the same time, shaping the landscape. So, olive groves are of great environmental and cultural importance.

Despite the difficulties related to the geomorphology of the Region, a significant number of farms and olive oil companies can be found throughout the territory. The management of the olive oil chain by-products from a circular perspective is, therefore, a priority objective. The principal contributor to waste generation are the leaves, deriving from both the pruning of trees and olives harvesting [63].

Nowadays, although olive leaves represent a promising source of bioactive compounds, the direct burning of the pruning residues is the preferred method of disposal in Liguria Region, which however entails economic costs and environmental risks.

With a view to recovering these residues, we evaluated the micro-morphological and anatomical features of olive leaves. This analysis represents an important starting point for the quality control process of plant by-products [64].

The micromorphological characterization of ‘Lavagnina’ leaf is in agreement with data obtained on other *Olea* cultivars by Bacelar et al. [65], Moreno-Alias et al. [66], Rahfeld [67] and Menezes et al. [68]. The diagnostic features of olive leaf are the presence of abundant scutiform trichomes, two zones of palisade parenchyma and individual or grouped elongated sclereids.

These characteristics are linked to the xerophytic nature of olive tree that has to cope with prolonged drought periods during the warm season of the Mediterranean-type climate, as highlighted by Marchioni et al. [69]. Indeed, according to Bosabalidis and Kofidis [70], the presence of the PPII and the high number of trichosclereids, which are involved in the sunlight distribution within the mesophyll, are a response to stress due to water shortage. In addition, the presence of phenolics in the stalk of scutiform trichomes, characteristic previously observed also by Rombaut et al. [71], could be implicated in the protective action of these trichomes against UV radiation.

Although the basic structure of olive leaf is shared between cultivars, according to Menezes et al. [68] and Žuna Pfeiffer et al. [72] there are some anatomical parameters that could differ, such as the midrib structure. For example, ‘Lavagnina’ leaf is similar to that of ‘Grapollo 561’ cultivar described by Menezes et al. [68], as they are both characterized by a bundle of sclerenchyma and collenchyma above the midrib, differently from other cultivars, e.g., ‘Neblina’ that is characterized by a larger bundle dominated by collenchyma [68].

In addition, we noticed that in ‘Lavagnina’ leaf the layer of PPII is not well defined, with loosely arranged cells not easily distinguishable from those of spongy parenchyma, differently from observations on leaves from other Spanish cultivars made by Moreno-Alias et al. [66]. In addition, the abaxial surface of ‘Lavagnina’ does not show a continuous layer of overlapping scutiform trichomes that completely hides the stomata, as instead was observed by Bacelar et al. [65] on two Portuguese cultivars. However, it remains difficult to determine whether these anatomical differences are the result of ecological adaptations, as the Ligurian olive groves facing the sea share similar pedo-climatic conditions. 

Extracts of leaves of different Mediterranean olive cultivars have been analyzed in various works, but relevant contributions on antioxidant activity of *O. europaea* ‘Lavagnina’ are lacking. Therefore, the present research investigated for the first time the phytochemical profile and biological activity of extracts from pruned leaves of this Ligurian olive. However, it is not easy to compare our results with those of other studies because of the heterogeneity in extracts preparation, tests used and data expressions.

In our work, the extraction of bioactive compounds was carried out using solvents of increasing polarity (PEE, CHE and ME) as well as the safe and food grade HAE.

HAE resulted the most suitable solvent for the extraction of phenolics and to ensure a high antioxidant activity. Phenolic compounds, flavonoids, secoiridoids, and secoiridoid glycosides are present in almost all the parts of *O. europaea* [73], but in significantly higher quantities in olive leaves [13]. Within the leaf, phenolics occur in the mesophyll and trichome stalks, as highlighted by Liakopoulos et al. [74], who also demonstrated that these structures differed chemically. For example, quercetin was detected in trichome layer but not in leaf mesophyll, suggesting that it could be a specific marker of this layer. Our results confirm the presence of phenolic compounds in trichome stalks and parenchyma, particularly in the multilayered palisade, as indicated by the blue-green staining with TBO.

Among the identified compounds, secoiridoids and flavonoids were the most abundant components. Secoiridoids, e.g., oleuropein and ligstroside, are unique to members of the Oleaceae family. They are iridoids derivatives that are generated by cleavage of the cyclopentane ring and present a phenolic or catechol moiety arising from the phenylpropanoid pathway. Oleuropein is the key component of secoiridoids, occurring in high amount particularly in olive leaves from which it can be easily extracted [19,75]. In a previous study, leaves of another Ligurian cultivar, *O. europaea* ‘Taggiasca’, were extracted with a mixture of 2-propanol:water and then this extract was partitioned to obtain an oleuropein-enriched fraction that was tested on mesothelioma cells. Results showed that this fraction displayed a significant antiproliferative effect, suggesting possible applications in the treatment of malignant mesothelioma [76].

In the literature, secoiridoids have been recognized as antioxidant, anti-inflammatory, and immunomodulatory compounds and, therefore, have been proposed as potential therapeutic agents for diseases driven by inflammation and reactive oxygen species (ROS), which can damage lipids, proteins and DNA [75,77].

Flavonoids, e.g., quercetin and luteolin-7-O-glucoside, consists of two aromatic rings connected by three carbons that typically form an oxygenated heterocycle. They occur either in the aglycone or glycosylated form [78]. These substances were reported for their potent antioxidant activity and protection against cardiovascular and carcinogenic diseases [19,77].

Other phenolic compounds of HAE were represented by both phenolic alcohols, as hydrotyrosol and its derivatives, and phenolic acids, as shikimic acid. They are among the simplest forms of phenolics, containing a phenolic ring and an organic carboxylic acid function, and exert beneficial effects in terms of antioxidation, anti-atherosclerosis, anti-carcinogenic and anti-inflammation [19,77]. Finally, HAE contained iridoids, including a wide group of cyclopentane [c] pyran monoterpenoids which have shown a wide range of bioactivities including anti-inflammatory, antibacterial, anti-carcinogenic, and antiviral [79]. Given the composition of HAE, as well as its antioxidant activities, this study underscores the potential of ‘Lavagnina’ leaves as a valuable source of bioactive compounds with applications in the medicinal, nutraceutical and cosmetic fields. In particular, since oxidative stress and inflammation are closely linked to cancer development and progression, HAE may display chemopreventive properties by protecting cells from oxidative damage and modulating inflammatory pathways. Further investigations, using specific in vitro and in vivo assays, will be useful to confirm and expand the indicated potential.

## 4. Materials and Methods

### 4.1. Plant Material

**Figure 5:**
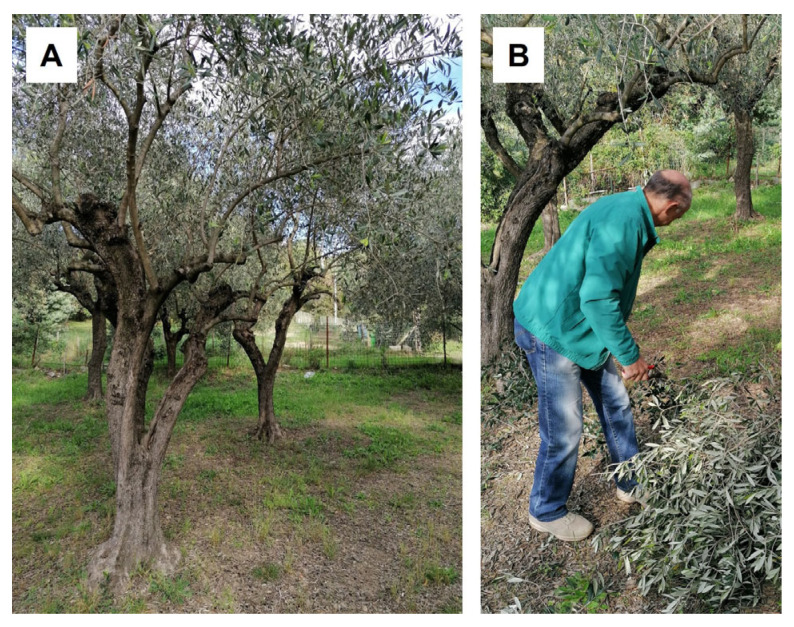
Trees of *Olea europaea* L. ‘Lavagnina’ (**A**), pruning process (**B**).

Olive pruning leaves of ‘Lavagnina’ cultivar (Figure 5A,B) were collected in October 2023 and June 2024 from the same trees and provided by “Società Cooperativa Agricola Olivicoltori Sestresi” based in Sestri Levante (Liguria, Italy). The leaves were air-dried before analysis. A voucher specimen (GDOR n. 63471) was deposited in the herbarium of the Natural History Museum Giacomo Doria of Genova (Italy).

### 4.2. Micro-Morphological and Anatomical Analyses

Mature leaves were fixed in a FineFIX working solution (Milestone SRL, Sorisole, Bergamo, Italy) and left overnight at 4 °C [80]. Then, small fragments deriving from the middle of the leaf were dehydrated in a graded alcohol series (70, 80, 90, and 100%) and embedded in Technovit 7100 (Heraeus Kulzer GmbH, Wehrheim, Germany). The samples obtained were finally cut into semiultrathin sections (8 μm) using a rotary microtome Leica RM 2155 (Leica Microsystems GmbH, Wetzlar, Germany). Sections were stained with the polychromatic dye TBO [81,82] or with Sudan Black [83] and finally observed under a Leica DM 2000 microscope (Leica Microsystems, Wetzlar, Germany) using a ToupCam Digital Camera, CMOS Sensor 3.1 MP resolution (ToupTek Photonics, Hangzhou, China). Polarized light was used to detect the presence of calcium oxalate crystals.

Small portions of the median zone of the leaves, after fixation, were also critical point-dried in CO_2_ (CPD, K850 2M Strumenti s.r.l., Rome, Italy), mounted on aluminum stubs, covered with a 10 nm layer of gold and observed under a VEGA3-Tescan-type LMU microscope (Apollo, Tescan USA Inc., Cranberry Twp, PA, USA), operating at an accelerating voltage of 20 kV.

For comparison, hand-made leaf sections of ‘Lavagnina’ and other two cultivars, ‘Leccino’ (from Sestri Levante, Eastern Liguria, GDOR. n. 63472) and ‘Taggiasca’ (from Imperia, Western Liguria, GDOR. n. 63473) were prepared and stained with TBO to reveal potential anatomical differences (see Supplementary Materials).

### 4.3. Extraction

A first sampling of ‘Lavagnina’ pruned leaves (October 2023, 800 g) was extracted using organic solvents of increasing polarity: petroleum ether 40–60° (PEE), chloroform (CHE) and methanol (ME). Since the biological assays indicated that only ME exhibited notable antioxidant properties, we repeated the sample collection (June 2024, 251.4 g) and performed extraction using food-grade 70% ethanol (HAE), given that methanol and ethanol have very similar solubility.

The extraction of olive leaves was carried out by maceration in glass flasks using 10 mL of solvent for each g of leaves. The flasks filled with leaves and solvent were stirred using a magnet. Each extraction cycle lasted 5 days and 3 extraction cycles were carried out to maximize the extraction. Once the extracts, derived from the use of the same solvent, were combined, the solvent was removed using a rotary evaporator and the extract was freeze-dried to remove residual water and stored in hermetically sealed falcons away from heat, light and humidity. The four extracts were then stored at 4 °C in dark glass bottles until analysis. The extraction yield for each extract was calculated as follows: Y (%) = (m/M) × 100, where m is the mass of the extracted residue (g), and M is the initial mass of the plant material (g).

### 4.4. Antioxidant Activity

#### 4.4.1. DPPH Assay

The antioxidant activity was determined using the stable 1,1-diphenyl-2-picrylhydrazyl (DPPH) radical method as reported by Brand-Williams and Coworkers [84] with some modifications. The analysis was performed in cuvettes by adding 25 μL of a solution of the extracts in MeOH to 975 μL of a DPPH solution (7.6 × 10^5^ M), which was prepared daily and kept in the dark to have a final volume of 1 mL in a straight-sided cuvette. Methanol alone was used as a blank, while 1 mL of DPPH solution (60 μM) was used as a control sample for a baseline measurement. After 45 min of incubation, the absorbance at 515 nm was measured in the spectrophotometer Thermo scientific Multiskan GO (Thermo Fischer Scientific, Vantaa, Finland). The percent inhibition of free radical formation by DPPH (I%) was calculated as follows:

I% = [1 − (A sample/A DPPH)] × 100

where A DPPH is the absorbance of the control reaction (containing all reagents except the test compound) and A sample is the absorbance of the test read at 515 nm after 45 min. The scavenging activity was expressed as the 50% effective concentration (EC50), which is defined as the sample concentration (μg/mL) that causes a 50% decrease in the DPPH absorbance. The experiments were carried out in triplicate, with triplicates obtained from the same extract, and the results were expressed as the mean ± standard deviation.

#### 4.4.2. ABTS Assay

The 2,20-azino-bis 3-ethylbenzothiazoline-6-sulfonic acid (ABTS) test was carried out following the method of Re and coworkers [85]. The radical ABTS (ABTS°+) was generated mixing an aqueous solution of ABTS (7 mM) and potassium persulfate (2.45 mM), that was left in the dark for 16 h at room temperature and then diluted with ethanol to an absorbance of 0.800 at 734 nm. An aliquot of 0.1 mL of each extract was mixed with 0.9 mL of diluted ABTS; then the mixture was incubated for 6 min. The absorbance was read at 734 nm. Trolox (6-hydroxy-2,5,7,8-tetramethylchroman-2-carboxylic acid) was dissolved in methanol at different concentrations and used as a reference standard. The results were expressed as the μM Trolox equivalent antioxidant capacity (TEAC) per gram of samples. The positive control was represented by a solution of the standard antioxidant, ascorbic acid, whose absorbance was measured under the same conditions as the samples studied. The experiments were carried out in triplicate, with triplicates obtained from the same extract, and the results were expressed as the mean ± standard deviation.

#### 4.4.3. FRAP Assay

The FRAP (Ferric Ion Reducing Antioxidant Power) assay was performed following the protocol of Benzie and Strain [86]. A FRAP reagent is a solution consisting of 23 mM acetate buffer (pH 3.6), 10 mM of tripyridyl triazine (TPTZ) in 40 mM of HCl, and 20 mM of FeCl_3_ (in a 10:1:1 ratio). Different concentrations of ferrous sulfate heptahydrate (FeSO_4_•7H_2_O), in the range 1–0.1 mM, were prepared to obtain the calibration curve. The reaction was carried out for each sample in a final volume of 272 μL in wells. The reaction mixture was incubated at 37 °C for 30 min in dark conditions. The absorbance of the blank, consisting of FRAP alone and monitored spectrophotometrically (Thermo scientific Multiskan GO, Thermo Fischer Scientific, Vantaa, Finland) at the wavelength of 593 nm, was subtracted from the absorbance of the FRAP with the sample to determine the FRAP value for each sample. The FRAP values were determined using the FeSO_4_•7H_2_O calibration curve and expressed as mmol Fe^2+^/g of dried extract. The experiments were carried out in triplicate, with triplicates obtained from the same extract, and the results were expressed as the mean ± standard deviation.

### 4.5. Chemical Analysis

The extracts were examined by LC-ESI-HR-MS, utilizing a Q Exactive: hybrid quadrupole-Orbitrap mass spectrometer (Thermo Fisher, Waltham, MA, USA), operating in negative ionization mode following Crescenzi and colleagues [87], with minor adjustments. LC-MS analysis was performed on a Luna 5 μm C18 100 Å (150 × 2 mm) column (Phenomenex, Aschaffenburg, Germany), at a flow rate of 0.2 mL/min. A binary mobile phase was employed (eluent A: H_2_O + 0.1% formic acid (99.9:0.1, *v*/*v*) and eluent B: H_3_CN + 0.1% formic acid (99.9:0.1, *v*/*v*). The HPLC program started at 5% B, reaching 95% B after 30 min; this level was maintained for an additional 5 min before returning to the starting percentage. The autosampler was set to inject 5 μL of each sample (1 mg/mL). The HESI source settings were as follows: spray voltage −2.5KV; S-Lens RF Level 50%; ion source temperature 300.01 °C; sheath and auxiliary gas flow (N_2_), 50 and 10; and sweep gas 0. The mass range for MS spectra acquisition was 50–1400 *m*/*z*. For the fragmentation study, a data-dependent acquisition was configured, fragmenting the precursor ions of the most intense peaks in the MS analysis with a collision energy of 30%. Xcalibur software version 2.2 was used for system control, data collection, and data processing.

Compound attribution was performed by comparison with the published literature and by evaluating MS/MS (MS^2^) fragmentation patterns. Specifically, we cross-checked accurate mass, isotopic distribution, plausible adducts, retention behavior, and—crucially—diagnostic product ions against reported spectra and fragmentation pathways for the proposed structures.

### 4.6. Statistical Analysis

Data were analyzed by a two-way ANOVA followed by Tukey’s post hoc test using Statistical Package for the Social Sciences (SPSS) version 13.0, 2004 (Chicago, IL, United States). The significance level has been set at 0.05.

## 5. Conclusions

Overall, the data collected in this study highlight the importance of recovering bioactive compounds from the pruned leaves of ‘Lavagnina’ cultivar, which has been scarcely investigated. Indeed, this by-product represents a valuable source of antioxidants potentially suitable for use in the medicinal, nutraceutical, and cosmetic fields. Notably, the high antioxidant activity (17.3 ± 0.6 μg/mL) of the HAE could exert a protective effect against DNA damage induced by free radicals, thereby potentially preventing mutagenesis and the onset of carcinogenesis. HAE is, therefore, a promising candidate for further in-depth studies to explore its potential chemopreventive activity in appropriate biological models and clinical trials. In this perspective, also toxicity tests will be conducted on this extract.

Future research will include pharmacognostic studies of the pruned leaves of different Italian *O. europaea* cultivars with the aim of better characterizing the anatomical differences among them and their phytochemical profile. This information could direct the harvesting of such residues in order to obtain extracts with high biological activity for new applications in human well-being. This approach also aligns with the principles of circular bioeconomy, promoting the recovery and valorization of agricultural wastes, such as pruned leaves, as a valuable resource for the development of sustainable and innovative natural products.

## Figures and Tables

**Table 1:** Antioxidant activity of *Olea europaea* L. ‘Lavagnina’ leaf extracts, expressed as mean ± SD of three experiments.

Extract	DPPH IC_50_^1^ (μg/mL)	FRAP mmol Fe^2+^ Equivalents/g Extract	ABTS mmol TEAC ^2^/g
PEE	>1000	-	>1000
CHE	79.4 ± 0.7 ^a^	1.4 ± 0.5 ^b^	0.9 ± 0.03 ^b^
ME	29.7 ± 0.6 ^b^	2.6 ± 0.3 ^b^	0.7 ± 0.05 ^b^
HAE	17.3 ± 0.6 ^c^	2.8 ± 0.7 ^b^	1.3 ± 0.04 ^b^
Trolox	3.21 ± 0.2 ^d^	84.2 ± 0.9 ^a^	-
Ascorbic acid	-	-	39.3 ± 0.8 ^a^

^1^ IC_50_ = concentration required to reduce the absorbance of the DPPH solution by 50%; ^2^ TEAC: trolox equivalent antioxidant activity. Trolox and ascorbic acid were used as reference standards. Means followed by different letters in the same column indicate that they are significantly different at *p* < 0.05, according to a two-way ANOVA followed by Tukey’s post hoc test. The letters have no intrinsic meaning but serve only to identify groups between which there are significant differences.

**Table 2:** Composition of ME and HAE of *Olea europaea* L. ‘Lavagnina’. The x indicates the presence of metabolite in the extracts.

No.	Family	Retention Time	Measured (*m*/*z*) [M-H]^−^	Molecular Formula	Δppm	Fragment	Fragment Formula	Fragment ion (*m*/*z*)	Δppm	Identification	ME	HAE
1	Alcohol sugar	1.72	181.0709	C_6_H_14_O_6_	1.190	[M-H_2_O-H]^−^	C_6_H_11_O_5_	163.0604	1.963	Mannitol	x	x
						[M-CH_6_O_2_-H]^−^	C_5_H_7_O_4_	131.0338	−0.65			
						[M-C_2_H_6_O_2_-H]^−^	C_4_H_7_O_4_	119.0339	0.04			
						[M-C_2_H_8_O_3_-H]^−^	C_4_H_5_O_3_	101.0232	−0.797			
2	Organic acid	1.87	195.0503	C_6_H_12_O_7_	0.411	[M-H_2_O-H]^−^	C_6_H_9_O_6_	177.0398	2.347	Gluconic acid		x
						[M-2H_2_O-H]^−^	C_6_H_7_O_5_	159.0289	0.756			
						[M-CH_6_O_3_-H]^−^	C_5_H_5_O_4_	129.0183	0.348			
						[M-C_2_H_8_O_4_-H]^−^	C_4_H_3_O_3_	99.0075	−1.217			
						[M-C_4_H_8_O_4_-H]^−^	C_2_H_3_O_3_	75.0075	−1.606			
3	Organic acid	2.48	191.0553	C_7_H_12_O_6_	1.547	[M-H_2_O-H]^−^	C_7_H_9_O_5_	173.0445	0.174	Quinic acid	x	x
						[M-2H_2_O-CO-H]^−^	C_6_H_7_O_3_	127.0388	−0.949			
						[M-2H_2_O-CO_2_-H]^−^	C_6_H_7_O_2_	111.0439	−0.955			
						[M-3H_2_O-CO_2_-H]^−^	C_6_H_5_O	93.0334	−1 412			
						[M-C_3_H_6_O_4_-H]^−^	C_4_H_5_O_2_	85.0283	−1 599			
4	Sugar	2.59	237.0616	C_8_H_14_O_8_	4.582	[M-C_3_H_8_O_4_-H]^−^	C_5_H_5_O_4_	129.0183	0.193	unknown (3-deoxy-D-manno-octulosonate)	x	x
						[M-C_5_H_10_O_5_-H]^−^	C_3_H_3_O_3_	87.0075	−1.385			
5	Secoiridoid	7.29	407.15652	C_17_H_28_O_11_	4.254	[M-C_8_H_16_O_9_-H]^−^	C_9_H_11_O_2_	151.07536	0.026	1-b-D-glucopyranosyl acyclodihydroelenolic acid		x
						[M-C_9_H_16_O_9_-H]^−^	C_8_H_11_O_2_	139.07545	0.675			
						[M-C_13_H_20_O_7_-H]^−^	C_4_H_7_O_4_	119.03346	−3.572			
6	Iridoid	7.38	199.0608	C_9_H_12_O_5_	3.617	[M-CO_2_-H]^−^	C_8_H_11_O_3_	155.0705	1.285	Hydroxylated product of the dialdehydic form of decarboxymethyl-elenolic acid		x
						[M-2CO_2_-H]^−^	C_7_H_11_O	111.0804	0.076			
						[M-C_7_H_8_O_3_-H]^−^	C_2_H_3_O_2_	59.0127	−1.286			
7	Phenolic group	7.48	315.1091	C_14_H_20_O_8_	5.160	[M-C_6_H_10_O_5_-H]^−^	C_8_H_9_O_3_	153.0549	1.564	Hydroxytyrosol glucoside	x	x
						[M-C_6_H_10_O_5_-H_2_O-H]^−^	C_8_H_7_O_2_	135.0440	−0.489			
						[M-C_7_H_12_O_6_-H]^−^	C_7_H_7_O_2_	123.0441	0.195			
						[M-C_11_H_14_O_5_-H]^−^	C_3_H_5_O_3_	89.0232	−0.792			
8	Secoiridoid	7.53	389.10925	C_16_H_22_O_11_	3.629	[M-C_6_H_10_O_5_-H]^−^	C_10_H_11_O_6_	227.0558	3.327	Oleoside (isomers)	x	x
						[M-C_6_H_10_O_5_-CO_2_-H]^−^	C_9_H_11_O_4_	183.0657	3.085			
						[M-C_8_H_10_O_9_-H]^−^	C_8_H_11_O_2_	139.0755	1.034			
						[M-C_8_H_12_O_10_-H]^−^	C_8_H_9_O	121.0648	0.318			
						[M-C_12_H_16_O_8_-H]^−^	C_4_H_5_O_3_	101.0233	−0.401			
						[M-C_13_H_16_O_8_-H]^−^	C_3_H_5_O_3_	89.0232	−1.129			
						[M-C_14_H_18_O_9_-H]^−^	C_2_H_3_O_2_	59.0127	−1.286			
9	Phenolic group	7.92	477.1620	C_20_H_30_O_13_	3.610	[M-C_14_H_20_O_8_-H]^−^	C_6_H_9_O_5_	161.0447	1.305	Hydroxytyrosol-diglucoside	x	x
						[M-C_12_H_20_O_10_-H]^−^	C_8_H_9_O_3_	153.0549	1.629			
						[M-C_12_H_20_O_10_-CH_2_O-H]-	C_7_H_7_O_2_	123.0440	−0.455			
10	Phenolic group	8.01	153.0549	C_8_H_10_O_3_	1.433	[M-CH_2_O-H]^−^	C_7_H_7_O_2_	123.0441	0.276	Hydroxytyrosol	x	x
						[M-C_2_H_2_O_2_-H]^−^	C_6_H_7_O	95.0492	0.722			
						[M-C_3_H_4_O_2_-H]^−^	C_5_H_5_O	81.0333	−2.732			
11	Secoiridoid	8.13	375.1301	C_16_H_24_O_10_	3.803	[M-C_6_H_10_O_5_-H_2_O-CO_2_-H]^−^	C_9_H_11_O_2_	151.0760	4.063	Loganic acid	x	x
						[M-C_8_H_10_O_9_-H]^−^	C_8_H_13_O	125.0958	−2.411			
						[M-C_12_H_16_O_6_-H]^−^	C_4_H_7_O_4_	119.0334	−3.824			
						[M-C_11_H_18_O_7_-H]^−^	C_5_H_5_O_3_	113.0232	−1.244			
						[M-C_12_H_18_O_7_-H]^−^	C_4_H_5_O_3_	101.0223	−3.866			
						[M-C_13_H_18_O_7_-H]^−^	C_3_H_5_O_3_	89.0230	−4.275			
						[M-C_13_H_20_O_8_-H]^−^	C_3_H_3_O_2_	71.0125	−3.040			
12	Iridoid	8.50	345.1195	C_15_H_22_O_9_	4.263	[M-C_6_H_10_O_5_-H]^−^	C_9_H_11_O_4_	183.0656	4.232	Aucubin	x	
						[M-C_6_H_10_O_5_-H_2_O-H]^−^	C_9_H_9_O_3_	165.0552	3.449			
						[M-C_11_H_14_O_5_-H]^−^	C_4_H_7_O_4_	119.0338	−0.716			
						[M-C_12_H_16_O_6_-H]^−^	C_3_H_5_O_3_	89.0233	−0.568			
13	Iridoid	8.93	215.0922	C_10_H_16_O_5_	3.766	[M-CO_2_-H]^−^	C_9_H_15_O_3_	171.1020	2.274	Aldehydic form of decarboxymethyl elenolic acid	x	x
						[M-CO_2_-H_2_O-H]^−^	C_9_H_13_O_2_	153.0912	1.266			
						[M-CO_2_-H_2_O-CO-H]^−^	C_8_H_13_O	125.0961	0.227			
14	Phenolic aldehyde derivative	8.96	313.0933	C_14_H_18_O_8_	4.683	[M-C_6_H_10_O_5_-H]^−^	C_8_ H_7_ O_3_	151.0393	2.048	Vanilloside		x
						[M-C_6_H_10_O_5_-CO-H]^−^	C_7_H_7_O_2_	123.0441	0.276			
						[M-C_11_H_14_O_5_-H]^−^	C_3_H_3_O_3_	87.0075	−1.615			
15	Iridoid	8.98	359.1353	C_16_H_24_O_9_	4.514	[M-C_6_H_10_O_5_-H]^−^	C_10_H_13_O_4_	197.0814	2.662	7-Deoxyloganic acid	x	x
						[M-C_6_H_10_O_5_- C_4_H_6_O-H]^−^	C_6_H_7_O_3_	127.0389	−0.713			
16	Secoiridoid	9.00	421.1724	C_18_H_30_O_11_	4.635	[M-C_9_H_18_O_9_-H]^−^	C_9_H_11_O_2_	151.075	−2.093	Oleoside methyl ester derivative		x
						[M-C_13_H_22_O_8_-H]^−^	C_5_ H_7_ O_3_	115.0388	−1.309			
17	Secoiridoid glycoside	9.04	257.1032	C_12_H_18_O_6_	4.960	[M-CO_2_-H]^−^	C_11_H_17_O_4_	213.1130	4.103	3-Hydroxymethyl-2.3-dihydro-5-(methoxycarbonyl)-2-methyl-2H-pyran-4-acetic methyl ester		x
						[M-C_3_H_6_O_4_-H]^−^	C_9_H_11_O_2_	151.0758	2.806			
						[M-C_9_H_14_O_3_-H]^−^	C_3_H_3_O_3_	87.0075	−1.730			
18	Secoiridoid	9.10	565.1779	C_23_H_34_O_16_	2.847	/	/	/	/	Elenolic acid dihexoside isomer	x	x
19		9.27	345.1558	C_16_H_26_O_8_	4.131	/	/	/	/	1,1,12,12-Dodecanetetracarboxylic acid		x
20	Secoiridoid	9.43	245.1031	C_11_H_18_O_6_	4.632	[M-H_2_O-CO_2_-H]^−^	C_10_H_15_O_3_	183.1020	2.289	Acyclodihydroelenolic acid	x	x
						[M-C_3_H_7_O_4_-H]^−^	C_8_H_11_O_2_	139.0752	−1.123			
21	Iridoid	9.57	389.1459	C_17_H_26_O_10_	3.846	[M-CO_2_-H]^−^	C_16_H_25_O_8_	345.1531	−3.547	Loganin	x	x
						[M-2CO_2_-H]^−^	C_15_H_25_O_6_	301.1654	2.673			
						[M-C_13_H_18_O_6_-H]^−^	C_4_H_7_O_4_	119.0338	−0.296			
						[M-C_13_H_20_O_7_-H]^−^	C_4_H_5_O_3_	101.0233	−0.500			
22	Phenolic aldehyde	9.99	151.0392	C_8_ H_8_O_3_	1.717	[M-CH_2_-H]^−^	C_7_H_5_O_3_	137.0237	2.623	Vanillin		x
						[M-CO-H]^−^	C_7_H_7_O_2_	123.0441	0.764			
						[M-CHO-H]^−^	C_7_H_6_O_2_	122.0363	0.729			
23	Iridoid	10.36	377.1459	C_16_H_26_O_10_	4.048	[M-C_6_H_12_O_6_-H]^−^	C_10_H_13_O_4_	197.0815	3.271	Lamiol	x	x
						[M-C_6_H_12_O_6_-CO_2_-H]^−^	C_9_H_13_O_2_	153.0913	1.854			
						[M-C_6_H_12_O_6_-C_6_H_6_-H]^−^	C_4_H_7_O_4_	119.034	0.964			
24	Secoiridoid	10.77	403.1250	C_17_H_24_O_11_	3.453	[M-C_6_H_12_O_6_-H]^−^	C_11_H_11_O_5_	223.0613	5.335	Elenolic acid glucoside (oleoside methyl ester)	x	x
						[M-C_8_H_14_O_7_-H]^−^	C_9_H_9_O_4_	181.0501	2.898			
						[M-C_6_H_12_O_6_-CO_2_-H]^−^	C_10_H_11_O_3_	179.0702	−0.283			
						[M-C_13_H_18_O_8_-H]^−^	C_4_H_5_O_3_	101.0233	−0.203			
						[M-C_14_H_18_O_8_ -H]^−^	C_3_H_5_O_3_	89.0232	−1.129			
25	Caffeoyl phenylethanoid derivatives	11.20	461.1672	C_20_H_30_O_12_	3.962	[M-C_13_H_18_O_6_-H]^−^	C_7_H_11_O_6_	191.0556	3.169	Decaffeoylverbascoside	x	x
						[M-C_15_H_20_O_7_-H]^−^	C_5_H_9_O_5_	149.0444	0.001			
						[M-C_15_H_24_O_9_-H]^−^	C_5_H_5_O_3_	113.0233	0.172			
26	Secoiridoid	11.47	525.1621	C_24_H_30_O_13_	2119	[M-C_8_H_8_O_2_-H]^−^	C_16_H_21_O_11_	389.1088	2.473	Demethyl oleuropein	x	x
						[M-C_14_H_20_O_8_-H]^−^	C_10_H_9_O_5_	209.0452	3.588			
						[M-C_14_H_18_O_9_-H]^−^	C_10_H_11_O_4_	195.0656	2.331			
						[M-C_14_H_20_O_8_-CO_2_-H]^−^	C_9_H_9_O_3_	165.0550	2.177			
						[M-C_14_H_20_O_8_-2CO_2_-H]^−^	C_8_H_9_O	121.0649	0.566			
27	Flavonoid	11.55	609.1468	C_27_H_30_O_16_	2.920	[M-C_6_H_18_O_2_-H]^−^	C_21_H_11_O_14_	487.0153	2.030	Rutin	x	x
						[M-C_11_H_20_O_9_-H]^−^	C_16_H_9_O_7_	313.0347	1.249			
						[M-C_12_H_20_O_9_-H]^−^	C_15_H_9_O_7_	301.0351	2.727			
						[M-C_12_H_21_O_9_-H]^−^	C_15_H_8_O_7_	300.0279	4.786			
						[M-C_13_H_22_O_11_-H]^−^	C_14_H_7_O_5_	255.0296	3.255			
						[M-C_19_H_26_O_11_-H]-	C_8_H_3_O_5_	178.9981	3.298			
						[M-C_20_H_26_O_12_-H]-	C_7_H_3_O_4_	151.0028	1.357			
28	Secoiridoid	11.65	555.1727	C_25_H_32_O_14_	3.203	[M-C_10_H_16_O_6_-H]^−^	C_15_H_15_O_8_	323.0771	3.021	10-Hydroxyoleuropein	x	x
						[M-C_14_H_20_O_9_-H]^−^	C_11_H_11_O_5_	223.0609	3.766			
						[M-C_15_H_24_O_10_-H]^−^	C_10_H_7_O_4_	191.0342	1.805			
						[M-C_17_H_24_O_11_-H]^−^	C_8_H_7_O_3_	151.0392	1.717			
29	Flavonoid	11.76	593.152	C_27_H_30_O_15_	3.175	[M-C_6_H_10_O_4_-H]^−^	C_21_H_19_O_11_	447.0936	3.092	Luteolin-7-O-rutinoside	x	x
						[M-C_12_H_20_O_9_-H]^−^	C_15_H_9_O_6_	285.0407	4.826			
30	Flavonoid	12.05	447.0936	C_21_H_20_O_11_	3.226	[M-C_6_H_10_O_5_-H]^−^	C_15_H_9_O_6_	285.0407	4.931	Luteolin-O-hexoside isomer	x	x
31	Secoiridoid	12.23	569.1882	C_26_H_34_O_14_	3.071	[M-C_9_H_10_O_3_-H]^−^	C_17_H_23_O_11_	403.1241	1.543	2′′-Methoxyoleuropein		x
						[M-C_9_H_10_O_3_-C_6_H_12_O_6_-H]^−^	C_11_H_11_O_5_	223.061	3.901			
32	Secoiridoid	13.66	539.1759	C_25_H_32_O_13_	0.543	[M-C_6_H_10_O_5_-H]^−^	C_19_H_21_O_8_	377.1253	5.876	Oleuropein	x	x
						[M-C_7_H_16_O_7_-H]^−^	C_18_H_15_O_6_	327.0865	0.628			
						[M-C_6_H_10_O_5_-C_4_H_6_O-H]^−^	C_15_H_15_O_7_	307.0826	4.594			
						[M-C_6_H_10_O_5_-C_4_H_6_O-CH_3_OH-H]^−^	C_14_H_11_O_6_	275.0561	4.128			
						[M-C_14_H_20_O_8-_H]^−^	C_11_H_11_O_5_	223.0610	3.856			
						[M-C_19_H_20_O_7_-H]^−^	C_6_H_11_O_6_	179.0550	−0.193			
33	Secoiridoid	14.40	583.2039	C_27_H_36_O_14_	3.066	[M-C_10_H_12_O_3_-H]^−^	C_17_H_23_O_11_	403.1261	6.480	Lucidumoside C	x	x
						[M-C_10_H_12_O_3_-C_6_H_12_O_6_-H]^−^	C_11_H_11_O_5_	223.0610	3.632			
						[M-C_17_H_24_O_11_-H]^−^	C_10_H_11_O_3_	179.0707	2.621			
						[M-C_19_H_29_O_11_-H]^−^	C_8_H_7_O_3_	151.0392	1.320			
34	Secoiridoid	14.83	523.1826	C_25_H_32_O_12_	2.977	[M-C_6_H_10_O_5_-H]^−^	C_19_H_21_O_7_	361.1298	4.626	Ligstroside (deoxy Oleuropein)	x	
						[M-C_6_H_10_O_5_-C_4_H_6_O-H]^−^	C_15_H_15_O_6_	291.0877	4.897			
						[M-C_6_H_10_O_5_-C_4_H_6_O_3_-H]^−^	C_15_H_15_O_4_	259.0979	5.344			
35	Simple phenol	15.64	201.1127	C_10_H_18_O_4_	2.757	[M-H_2_O-H]^−^	C_10_H_15_O_3_	183.1020	2.398	Sebacic acid		x
						[M-CO_2_-H]^−^	C_9_H_17_O_2_	157.1226	1.996			
						[M-H_2_O-CO_2_-H]^−^	C_9_H_15_O	139.1119	0.851			
36	Flavonoid	15.98	285.2074	C_15_H_10_O_6_	4.510	[M-C_3_H_2_O_3_-H]^−^	C_12_H_7_O_3_	199.0395	2.911	Luteolin	x	x
						[M-C_3_O_2_-C_2_H_2_O-H]^−^	C_10_H_7_O_3_	175.0392	1.425			
						[M-C_8_H_6_O_2_-H]^−^	C_7_H_3_O_4_	151.0029	2.284			
						[M-C_7_H_4_O_4_-H]^−^	C_8_H_5_O_2_	133.0283	−0.646			
						[M-C_13_H_6_O_4_-H]^−^	C_2_H_3_O_2_	59.0126	−3.150			
37	Flavonoid	16.14	301.0358	C_15_H_10_O_7_	4.953	[M-CO-H]^−^	C_14_H_9_O_6_	273.0406	4.598	Quercetin	x	x
						[M-C_7_H_6_O_2_-H]^−^	C_8_H_3_O_5_	178.9980	2.907			
						[M-C_8_H_6_O_3_-H]^−^	C_7_H_3_O_4_	151.0029	1.887			
38	Flavonoid	17.60	269.0458	C_15_H_10_O_5_	5.055	[M-C_8_H_6_O-H]^−^	C_7_H_3_O_4_	151.0027	0.695	Apigenin		x
						[M-C_7_H_4_O_2_-H]^−^	C_8_H_5_O_3_	149.0234	0.802			
						[M-C_7_H_4_O_4_-H]^−^	C_8_H_5_O	117.0332	−2.233			
						[M-C_8_H_6_O-CO_2_-H]^−^	C_6_H_3_O_2_	107.0127	−0.148			
39	Flavonoid	17.90	299.0562	C_16_H_12_O_6_	4.098	[M-CH_3_-H]^−^	C_15_H_8_O_6_	284.0329	4.685	Diosmetin		x
						[M-C_2_H_4_-H]^−^	C_14_H_7_O_6_	271.0450	4.596			
						[M-CH_3_-CO-H]^−^	C_14_H_8_O_5_	256.0369	0.958			
40	Secoiridoid	18.73	377.1245	C_19_H_22_O_8_	3.834	[M-CH_3_OH-H_2_O-H]^−^	C_18_H_15_O_6_	327.0869	1.912	Oleuropein aglycone		x
						[M-C_4_H_6_O-H]^−^	C_15_H_15_O_7_	307.0825	4.106			
						[M-C_4_H_6_O-CH_3_OH-H]^−^	C_14_H_11_O_6_	275.0571	7.582			
						[M-C_11_H_16_O_5_-H]^−^	C_8_H_5_O_3_	149.0236	1.674			
						[M-C_12_H_14_O_5_-H]^−^	C_7_H_7_O_3_	139.0391	0.787			
						[M-C_13_H_14_O_7_-H]^−^	C_6_H_7_O	95.0491	−0.857			
41	Phenolic acid	22.34	173.0448	C_7_H_10_O_5_	1.850	[M-H_2_O-H]^−^	C_7_H_7_O_4_	155.0342	1.772	Shikimic acid	x	x
						[M-CO-H]^−^	C_6_H_9_O_4_	145.0496	0.515			
						[M-CO_2_-H]^−^	C_6_H_9_O_3_	129.0547	0.77			
42	Dicarboxylic acid	23.71	285.2074	C_16_H_30_O_4_	4.888	[M-H_2_O-H]^−^	C_16_H_27_O_3_	267.1969	5.235	Hexadecanedioic acid	x	x
						[M-H_2_O-CO_2_-H]^−^	C_15_H_27_O	223.2066	4.158			
						[M-C_6_H_14_O_3_-H]^−^	C_10_H_15_O	151.1112	−3.651			
43	Sugar	28.66	259.0825	C_11_H_16_O_7_	4.982	[M-C_2_H_2_O-H]^−^	C_9_H_13_O_6_	217.0701	−2.739	1.5-Anhydroxylitol	x	x
						[M-C_5_H_6_O_3_-H]^−^	C_6_H_9_O_4_	145.0494	−0.588			
						[M-C_6_H_8_O_4_-H]^−^	C_5_H_7_O_3_	115.039	0.603			

## Data Availability

The data presented in this study are available in this published paper.

## Supplementary Materials

The following supporting information can be downloaded at: https://www.mdpi.com/article/10.3390/molecules30173605/s1, Figure S1: Anatomical differences in leaves of different cultivars of *Olea europaea* L. are shown: ‘Lavagnina’ (A), ‘Taggiasca’ (B), ‘Leccino’ (C). The arrangement of vessels in the main vein assumes a different shape depending on the cultivar, and the main differences are represented by the thickening of the bundle of mechanical tissues located above the central vein (white arrows). Indeed, ‘Lavagnina’ (A) and ‘Taggiasca’ (B) are both characterized by a bundle of sclerenchymatic fibers and some collenchymatous cells, although it is slightly less developed in ‘Taggiasca’. In contrast, ‘Leccino’ (C) shows a larger bundle, composed mainly of collenchyma beginning to lignify.
