# Supplementary material for: Pharmacognosy and Antioxidant Activity of Pruned Leaves from the Unexplored Olea europaea L. ‘Lavagnina’ (Liguria, Italy)
Source: Molecules. 2025 Sep 3;30(17):3605. doi: 10.3390/molecules30173605 (PMC12430430; doi:10.3390/molecules30173605)

Figure S1 – Anatomical differences in leaves of different cultivars of *Olea europaea* L. are shown: 'Lavagnina' (A), 'Taggiasca' (B), 'Leccino' (C). The arrangement of vessels in the main vein assumes a different shape depending on the cultivar, and the main differences are represented by the thickening of the bundle of mechanical tissues located above the central vein (white arrows). Indeed, 'Lavagnina' (A) and 'Taggiasca' (B) are both characterized by a bundle of sclerenchymatic fibers and some collenchymatous cells, although it is slightly less developed in 'Taggiasca'. In contrast, 'Leccino' (C) shows a larger bundle, composed mainly of collenchyma beginning to lignify.

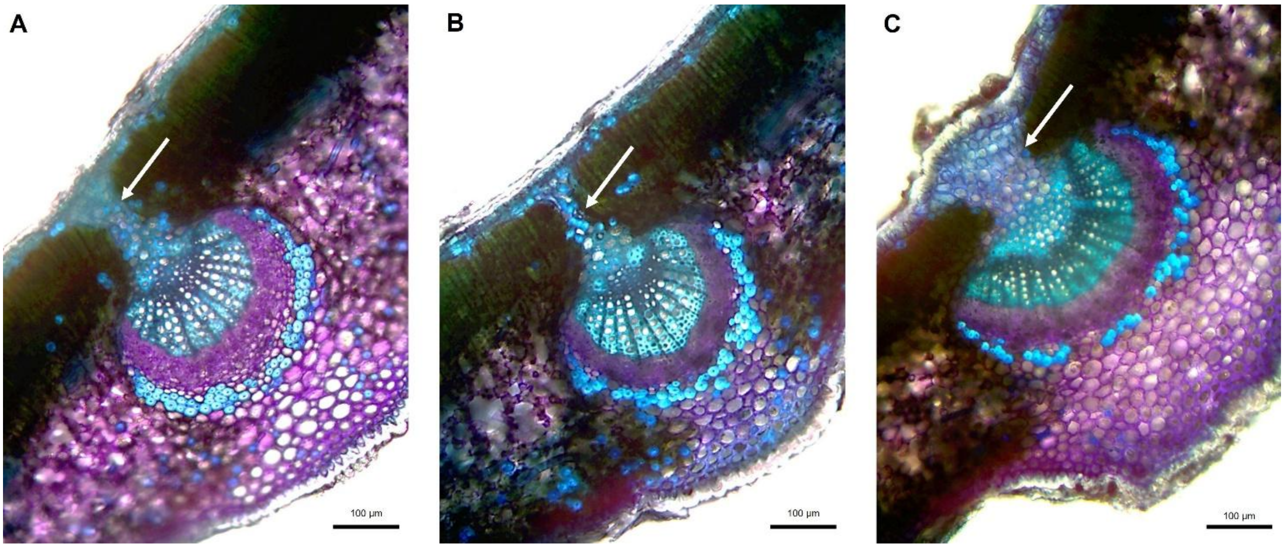

Supplement: Supplementary file 1 [file molecules-30-03605-s001.zip › Figure S1.pdf]
